# Supplementary material for: Polyketide synthase gene clusters in the ericoid mycorrhizal fungus Oidiodendron maius: genome-wide prediction, expression profiling, and functional characterization of knockout mutants
Source: Front Plant Sci. 2026 Sep 16;17:1924430. doi: 10.3389/fpls.2026.1924430 (PMC13623903; doi:10.3389/fpls.2026.1924430)
Supplement: Supplementary file 1 [file SupplementaryFile1.zip › Supplementary Materials/Supplementary Folder 1.zip!/Oidma1_AssemblyScaffolds_Repeatmasked/knownclusterblast/cluster47/jgi.p_Oidma1_60806_mibig_hits.html]

| MIBiG Protein | Description | MIBiG Cluster | MiBiG Product | % ID | % Coverage | BLAST Score | E-value |
| --- | --- | --- | --- | --- | --- | --- | --- |
| AMR44275.1 | zinc\_finger\_protein | BGC0001398\_c2 | Phomopsins\_biosynthetic\_gene\_cluster | 63 | 110.112359551 | 520 | 1.4e-147 |
| EAL89341.2 | C6\_transcription\_factor\_(AflR),\_putative | BGC0001403 | Trypacidin\_biosynthetic\_gene\_cluster | 37 | 20.6741573034 | 50 | 7.2e-06 |
